# Irreversible electroporation for pancreatic cancer using intraprocedural cone-beam computed tomography fusion navigation: a case report

**DOI:** 10.1186/s13256-021-03152-2

**Published:** 2021-11-25

**Authors:** Sriram Rao, Thanh-Lan Bui, Ali Sasani, Ben Sadeghi, Anvesh Macherla, Roozbeh Houshyar, Nadine Abi-Jaoudeh

**Affiliations:** Department of Radiological Sciences, University of California Irvine, 101 The City Drive South, Route 140, Orange, CA 92868 USA

**Keywords:** Locally advanced pancreatic cancer, Irreversible electroporation, Cone-beam computed tomography, Case report

## Abstract

**Background:**

Patients diagnosed with locally advanced pancreatic cancer are usually not eligible for surgical resection because of significant vascular involvement. Stereotactic body radiation therapy and chemotherapy are the treatments recommended by the National Comprehensive Cancer Network criteria. For patients who do not respond to or tolerate stereotactic body radiation therapy and/or chemotherapy, a new option is irreversible electroporation. Irreversible electroporation is a nonthermal minimally invasive ablation technique that uses electrical pulses to induce apoptosis of tumor cells without damage to the extracellular matrix, thus preserving ducts and vessels. Irreversible electroporation requires very precise needle placement, which has limited its ubiquitous use. Intraprocedural cone-beam computed tomography with navigation can be fused with previous imaging to provide real-time tumor navigation capabilities during the procedure to allow for more accurate needle placement and treatment. Here, we present a patient who underwent percutaneous irreversible electroporation with intraprocedural cone-beam computed tomography fusion guidance to treat his pancreatic cancer.

**Case presentation:**

The patient, an 88-year-old White male, initially presented with abdominal pain, and was ultimately diagnosed with locally advanced pancreatic cancer. He has an excellent performance status and no other comorbidities. He was started on chemotherapy and radiation therapy, with good response. However, continued vascular involvement of the tumors precluded him from safe surgical resection. The patient underwent irreversible electroporation with intraprocedural cone-beam computed tomography fusion navigation. The primary lesion demonstrates no residual tumor, and the soft tissue involvement of the adjacent vasculature has stabilized.

**Conclusions:**

Although not curative on its own, irreversible electroporation holds promise as a treatment option for patients with locally advanced pancreatic cancer to increase downsizing to curative surgery or increase quality of life. Cone-beam computed tomography navigation can improve irreversible electroporation by providing guidance during needle guidance. Image fusion with previous advanced imaging can improve lesion visualization and targeting, thereby improving the effectiveness of irreversible electroporation.

## Background

Pancreatic cancer is the seventh leading cause of cancer death in men and women worldwide [1]. Nearly half of patients who present with symptoms are diagnosed with metastatic disease because it is asymptomatic in its early stages. There are no curative treatment options for patients with metastatic disease. In contrast, in patients with early disease, surgical resection is known to increase overall survival and has the best prognosis. Patients downstaged from inoperable locally advanced disease to surgical candidates also experience the same benefits as patients who are surgical candidates at presentation [2]. Ideally, every nonmetastatic pancreatic cancer patient should undergo surgical resection. However, for patients with locally advanced pancreatic cancer [that is, focal disease that encases more 180° of the superior mesenteric artery (SMA) and/or superior mesenteric vein (SMV) without distant metastasis], safe surgical resection is challenging due to vascular complications.

Due to the abundance of vascular structures in the pancreatic region, irreversible electroporation (IRE) therapy has the potential to be the ideal treatment modality to target tumor cells in patients with locally advanced pancreatic cancer (LAPC) [3]. IRE is a nonthermal ablative method that can be applied successfully to areas around blood vessels and other vital structures without causing structural changes to the extracellular matrix, thus preserving the architecture of the ducts and vessels. IRE is also not subject to the heat sink effect commonly observed in other heat-based ablative techniques [4, 5].

Cone-beam computed tomography (CBCT) is an imaging modality that allows for near-real-time volumetric data acquisition in the interventional radiology suite [6] and has been employed for intraprocedural imaging and anatomical planning. CBCT has previously been described and used for procedures such as percutaneous tumor ablations and biopsies [7]. Recent advances have allowed for intraprocedural CBCT data to be fused with prior imaging, traditional computed tomography (CT), magnetic resonance imaging (MRI), or positron-emission tomography/computed tomography (PET/CT), to enable more informed real-time navigation during image-guided interventions [8].

In this case report, we present a patient with LAPC who was treated with IRE using CBCT fusion and navigation technology. His procedure employed intraprocedural CBCT fusion with preprocedural PET/CT for localization of his pancreatic cancer and navigation for needle placement of his IRE probes.

## Case presentation

**Fig. 1:**
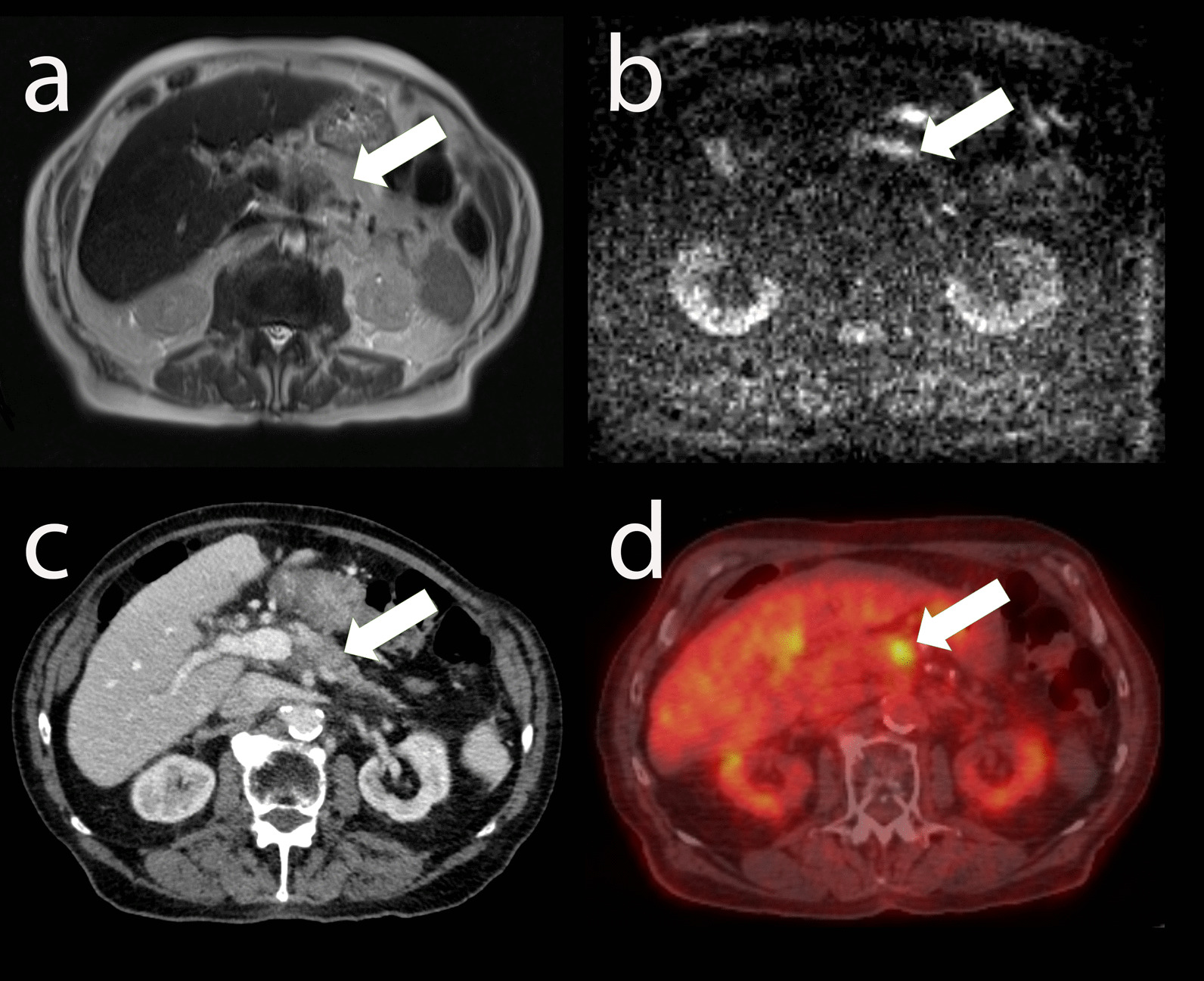
Axial T2 (**a**) and high *b*-value diffusion-weighted imaging (**b**) showing a lesion in the pancreatic body (arrows) prior to irreversible electroporation. Axial CT (**c**) showing a hypoenhancing soft tissue lesion (arrow) in the body of the pancreas that encases the mesenteric root vasculature and corresponds to a fluorodeoxyglucose (FDG)-avid lesion (arrow) on PET/CT (**d**)

The patient was an 88-year-old White male, with history of prostate cancer status post-prostatectomy, hypertension, and chronic kidney disease, who presented with mid-abdominal pain lasting over 2 months. Abdominal ultrasound with contrast revealed an echogenic mass in the pancreas. Subsequent MRI of the abdomen demonstrated a 5.3 × 4.2 cm lesion in the pancreatic body concerning for pancreatic adenocarcinoma (Fig. 1). This lesion demonstrated restricted diffusion with associated upstream pancreatic ductal dilation. Additionally, the mass encased the portal confluence (with severe narrowing of the splenic vein), distal celiac axis, proximal common hepatic artery, and splenic artery. No metastatic lesions were identified in the abdomen. At this time, carbohydrate antigen 19-9 (CA 19-9) was 1671 U/mL (reference range < 35 U/mL), amylase was 87 U/L (reference range 29–103 U/L), and lipase was 29 U/L (reference range 11–82 U/L).

Given the imaging findings, the patient was diagnosed with stage III locally advanced pancreatic adenocarcinoma and started on chemotherapy (Gemzar and Abraxane) and a course of stereotactic body radiation therapy. Over the next 12 months, CT imaging of the abdomen and chest demonstrated a response to therapy with a size reduction of the pancreatic mass to 1.4 × 0.9 cm and no signs of metastases. However, at 12 months post therapy initiation, the patient was not considered to be a candidate for tumor resection because of the continued unchanged soft tissue encasement of the nearby vasculature, presumed to be persistent tumor involvement. Sixteen months after initiation of therapy, PET/CT revealed a metabolically active lesion at the site of the primary pancreatic tumor. The patient was then recommended for IRE treatment for his pancreatic cancer. Leading to the procedure, CA 19-9 was 196 U/mL, amylase was 76 U/L, and lipase was 29 U/L. The lesion is not readily visible on unenhanced CT or ultrasound, only venous phase of contrast-enhanced CT.

**Fig. 2:**
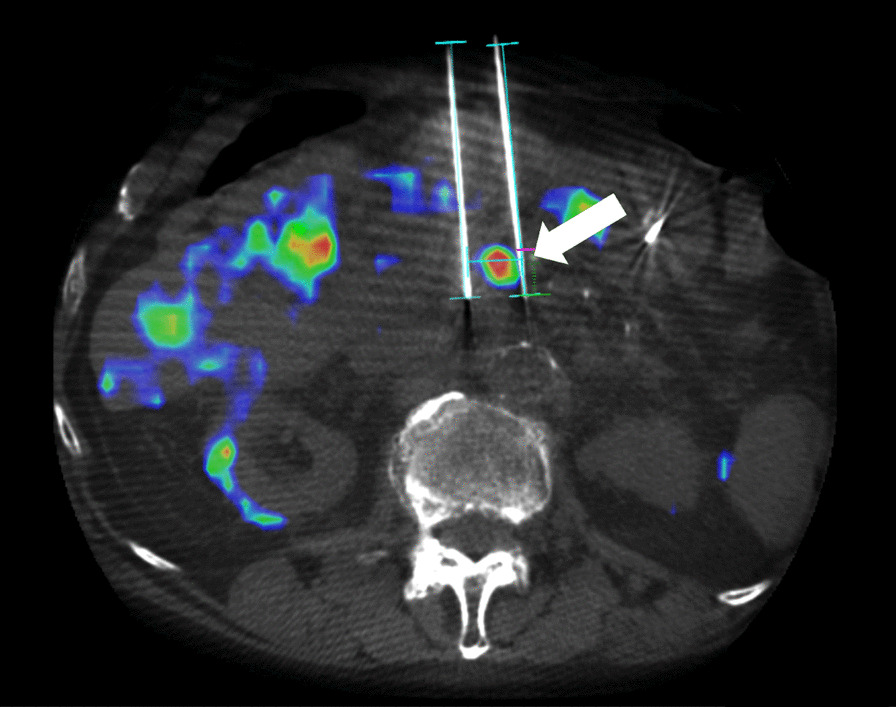
Intraprocedural fused cone-beam CT with preprocedural PET allows for accurate positioning of the IRE electrodes adjacent to the pancreatic body tumor (arrow)

Therefore, the patient’s previous PET/CT scan (Fig. 1) was imported into the 3D image analysis workstation (Xperguide Philips Healthcare, Best, The Netherlands). CBCT imaging alone did not show the lesion, but fusion with PET/CT enabled targeting of the tumor area. Skin entry points and paths were chosen for each of three IRE needles per manufacturer’s recommendation. The needles were placed approximately 1.5 cm from each other, as confirmed by CBCT imaging throughout placement (Fig. 2). After placement, the IRE probes were unsheathed by 1.5 cm, and the lesion was treated for two rounds of 90 electrical pulses each. The wattage was increased in between the two rounds.

**Fig. 3:**
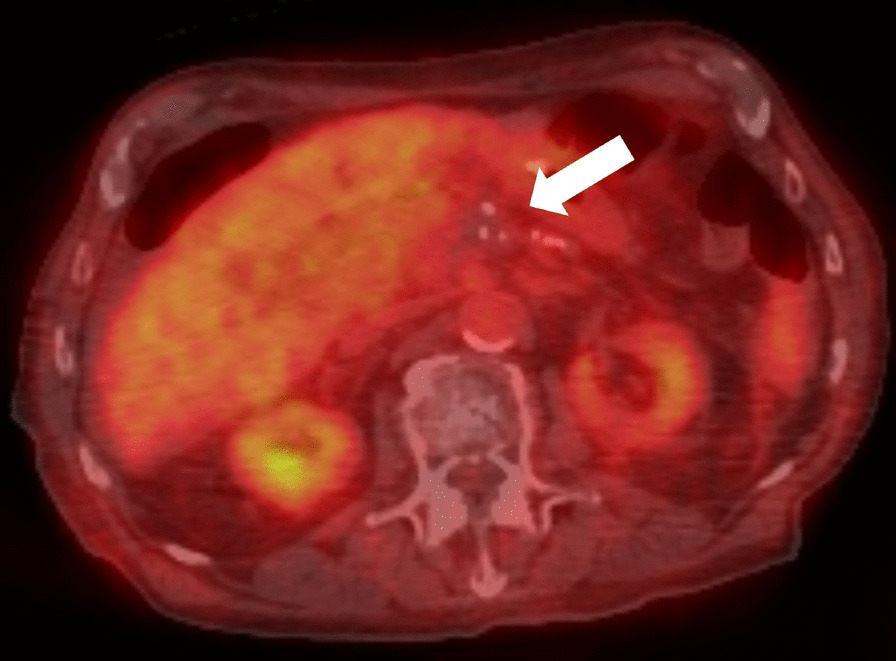
Postprocedural PET/CT demonstrating no residual FDG activity in the treatment site (arrow)

Postprocedurally, an expected but small amount of pneumoperitoneum was observed on CBCT. On follow-up PET/CT, no new or residual fluorodeoxyglucose (FDG) avidity was noted in the ablation zone (Fig. 3). Two weeks post-procedure, CA 19-9 was 266 U/mL, amylase was 64 U/L, and lipase was 10 U/L. Six months post-procedure (18 months from initial diagnosis), MRI scans have confirmed the presence of soft tissue surrounding the adjacent vasculature, which is nonspecific and may reflect post-treatment change since the PET was negative.

## Discussion

Until recently, a diagnosis of LAPC precluded patients from candidacy for surgical intervention because of the risks posed. Options for treatment were limited to stereotactic body radiation therapy, external beam radiation, and ethanol injections. However, these techniques present their own risks of collateral damage to adjacent vital structures and are therefore not always viable treatment options [9].

Given the nonthermal nature of IRE and its ability to preserve extracellular matrix, patients with LAPC are considered the ideal candidates for this intervention. Studies have shown that IRE can be used in conjunction with surgery and chemotherapy to optimize tumor margin accentuation and yield improvements in quality of life, progression-free survival, and overall survival for patients who meet diagnostic criteria for LAPC [10].

Percutaneous IRE of the pancreas is typically guided by ultrasound and/or CT fluoroscopy. Using either technique, the lesion in question may not be readily seen intraprocedurally, but rather only visible on the prior CT, MRI, or PET/CT scans. Also, needle placement is extremely challenging as they need to be parallel and within 1–2 cm of each other. In this case, the lesion was best localized on prior PET/CT. When intraprocedural imaging cannot visualize the lesion, targeting becomes challenging. Therefore, fusion of CBCT with the patient’s past PET/CT allowed his tumor to be targeted and enabled more accurate placement of the IRE needles due to navigation.

## Conclusions

Historically, patients with LAPC have not been considered surgical candidates; although, if downstaged to surgery, they can experience the same benefits in overall survival as patients who are surgical candidates at presentation. IRE, a minimally invasive nonthermal targeted procedure, presents a promising treatment option to maximize the chances for surgical resection and/or overall survival. Intraoperative CBCT navigation can aid precise needle placement, while CBCT fusion with prior imaging may improve targeting.

## Data Availability

Not applicable.

## Abbreviations

LAPC: Locally advanced pancreatic cancer
IRE: Irreversible electroporation
CBCT: Cone-beam computed tomography
MRI: Magnetic resonance imaging
CT: Computed tomography
PET/CT: Positron emission tomography/computed tomography
EUS: Endoscopic ultrasound
ERCP: Endoscopic retrograde cholangiopancreatography
FNA: Fine needle aspiration
SBRT: Stereotactic body radiation therapy
SMA: Superior mesenteric artery
SMV: Superior mesenteric vein
CA 19-9: Carbohydrate 19-9
